# Comparative Analysis of Graft Survival in Older and Younger Kidney Transplant Recipients: A Single-Center Cohort Study

**DOI:** 10.3390/jcm14248953

**Published:** 2025-12-18

**Authors:** Adolfo González Serrano, Ricardo José Guldris García, Gonzalo Gómez Marqués, Mercedes Ruiz Hernández, Enrique Carmelo Pieras Ayala

**Affiliations:** 1 Department of Urology, Hospital Universitari Son Espases, 07120 Palma, Spain; 2 Research Group in Nephro-Urological Diseases, Health Research Institute of the Balearic Islands (IdISBa), 07120 Palma, Spain; 3 Department of Nephrology, Hospital Universitari Son Espases, 07120 Palma, Spain

**Keywords:** aged, geriatrics, graft survival, kidney transplantation, transplant recipients

## Abstract

**Background/Objectives:** We hypothesized that older recipients have a higher rate of kidney graft failure compared to younger recipients. Thus, we assessed 60-month kidney graft failure (KGF) among deceased donor recipients aged 65 years or older and compared it with that of younger recipients. **Methods:** A single-center, retrospective cohort study was conducted at Son Espases University Hospital in Palma, Spain, including all consecutive deceased donor kidney transplant recipients from 2011 to 2021. The primary outcome was 60-month KGF, analyzed using the cumulative incidence function (CIF). A multivariable semi-parametric Fine and Gray model was used to estimate the subhazard of KGF in older versus younger recipients, adjusting for variables associated with recipients aged 65 years or older, including KGF and overall survival. **Results:** The study included 618 recipients, with a median age (interquartile range) of 58 years (47–66 years); of these, 187 (30%) were aged 65 years or older, and 498 (81%) received grafts from donors after brain death. The 60-month CIF (95% confidence interval) of KGF for the entire cohort was 12% (9.1–15). Candidate variables for multivariable analysis included recipient sex, body mass index, donor age, presence of hypertension or diabetes, donor sex, length of hospital stay, cold ischemia time, donor type, multiple renal veins, and Clavien-Dindo grade ≥ 3 complications. After adjustment, KGF risk did not significantly differ between age groups (sHR: 0.75; 95% CI: 0.41–1.38; *p* = 0.36). **Conclusions:** Despite having worse baseline characteristics, receiving lower-quality grafts, and experiencing a higher incidence of postoperative complications, we observed comparable 60-month kidney graft survival in older recipients relative to younger ones. These findings support the viability of kidney transplantation in well-selected older patients.

## 1. Introduction

The world’s population is aging, and a significant demographic shift is anticipated in the coming years [1].

Population aging leads to an increased risk of chronic diseases such as chronic kidney disease (CKD), and growth in the number of individuals at risk of developing it [2]. This demographic and epidemiological transition has led to the emergence of kidney diseases as one of the 10 most common causes of death worldwide and one of the major causes of years of life lost (YLL) [3,4]. Furthermore, besides age, some of the primary risk factors closely associated with CKD, such as hypertension, obesity, and diabetes, are also among the leading causes of YLL [4,5].

Patients aged ≥65 years represent 55% of patients starting renal replacement therapy (RRT) for CKD, and account for 42% of prevalent patients in RRT among European countries [6]. Among RRT, kidney transplantation (KT) represents the most cost-effective alternative due to improvements in survival, cost reduction, and quality of life [7,8]. However, older adults face longer waiting times and constitute the fastest-growing segment of the population among individuals on the KT waiting list [9]. Additionally, recent evidence has demonstrated that KT outcomes in this population are inferior to those in younger patients. Artiles et al. showed that 5-year kidney graft failure (KGF) was higher in patients aged ≥70 than in younger patients (Relative Risk, 1.37; 95% CI, 1.14–1.65) [10].

Therefore, our study aimed to assess 60-month KGF in patients aged ≥65 years and compare it with that of younger patients within a consecutive series of deceased donor kidney transplants at a single center.

## 2. Materials and Methods

### 2.1. Patients and Setting

This was a retrospective, single-center cohort study conducted at Son Espases University Hospital in Palma, Spain, including all consecutive deceased donor kidney transplant patients from 2011 to 2021. All patients were aged 18 years or older and underwent a Taguchi ureteroneocystostomy. This study received approval from an independent ethics committee (IB 5288/23 PI).

### 2.2. Outcome Ascertainment

The primary outcome was 60-month KGF, defined as the interval between KT and return to dialysis or need for another transplantation. The secondary outcome was overall survival (OS), defined as the interval between KT and death from any cause.

### 2.3. Follow-Up

The follow-up time started on the date of KT and ended on the date of KGF, the date of data extraction (31 December 2022), death, or, for censored patients, the date of last follow-up, whichever occurred first.

### 2.4. Covariates

The World Health Organization (WHO) classifies individuals aged 60 years and above as older adults. In the context of KT, a threshold age of 65 years has often been applied [11]. Thus, older patients were defined as those aged 65 years and over.

We considered the following receptor characteristics: sex, age, body mass index (BMI), existence of previous KT, hypertension, and diabetes mellitus. Donors’ variables included age, sex, type of donor (donors after brain death (DBD) and donors after circulatory death (DCD)), number of arteries, veins, and ureters. Operative variables included cold ischemia time, length of stay, and the occurrence of grade 3 or higher Clavien-Dindo complications.

### 2.5. Statistical Analysis

Summary statistics were used to describe baseline demographic and clinical characteristics. Frequencies and percentages were used to describe categorical variables, and the median and interquartile range (IQR) were used to describe continuous variables.

Differences between older and younger patients were analyzed using the chi-squared or Fisher’s exact test for categorical variables, and either the *t*-test or the Wilcoxon test for continuous variables, depending on the data distribution. We also performed a logistic regression analysis to identify factors associated with patients aged ≥65 years, and crude odds ratios (OR) and their 95% confidence intervals (CI) were reported.

As death and KGF are not independent because death prevents KGF, death from any cause before KGF was considered a competing event. Under these assumptions, KGF tends to be overestimated using a parametric method, such as the Kaplan-Meier estimator, because graft survival information is lost in patients who die with functional grafts. So, death-censored graft failure (DCGF) has been proposed to address this issue [12]. However, DCGF also leads to upwardly biased estimates of the risk of graft failure [13]. Thus, we calculated the cumulative incidence function of graft failure (CIF) to address these issues. CIF between groups was compared using Gray’s test. We used a semi-parametric Fine and Gray model to estimate differences in CIF and crude subhazard ratios (sHR) of KGF, which were quoted with their 95% CI [14]. OS was analyzed using the Kaplan-Meier estimator. The log-rank test assessed differences in OS among groups. Factors associated with survival were assessed using a Cox regression to estimate crude HR and their 95% CI.

Candidate variables for multivariable analysis were those associated at *p* < 0.2 with age-related differences in patients aged ≥65 years, and with KGF and OS in our cohort, and those identified in the previous meta-analysis and available in our dataset [15]. A multivariable Fine and Gray model was specified to estimate adjusted subhazard ratios (sHR) and their 95% CI.

The proportional hazards assumption was assessed using the Schoenfeld residuals test.

As other studies have used a 70-year threshold to define older patients, we performed sensitivity analyses using this threshold [10].

All statistical tests were two-sided using an alpha threshold error for significance of <0.05. All statistical analyses were performed using Stata software (version 14.2, StataCorp, College Station, TX, USA) and RStudio (version 2025.09.1).

## 3. Results

### 3.1. Demographic Characteristics of Participants

We included 618 participants. The median age (IQR) was 58 (47–66), 187 (30%) were patients aged ≥65 years, 412 (67%) were male, and 498 (81%) received grafts from DBD. Other baseline characteristics are described in Table 1.

### 3.2. Differences Between Age Groups

Compared to younger patients, older patients were more frequently males, had a higher body mass index (BMI; mean: 28 vs. 27; *p* = 0.047), received grafts from older donors (mean age: 66 vs. 51 years; *p* < 0.001), and had higher rates of hypertension (92% vs. 84%; *p* = 0.006) and diabetes (42% vs. 23%; *p* < 0.001), and experienced more complications of grade ≥ 3 (33% vs. 21%; *p* = 0.001) (Table 2).

### 3.3. Kidney Graft Failure

**Figure 1:**
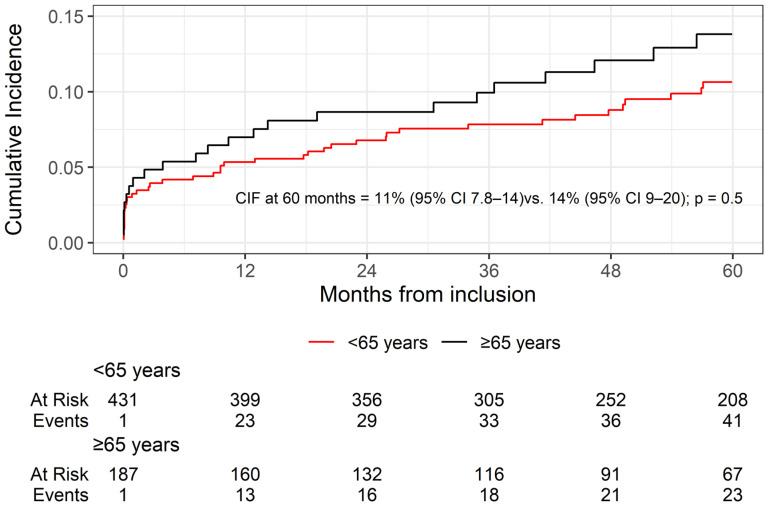
Comparison of the cumulative incidence functions between patients aged ≥65 years and younger over a 60-month follow-up period. CIF: Cumulative Incidence Function, 95% CI: 95% confidence interval.

Follow-up time was available for all participants. The median (IQR) length of follow-up was 53 months (28–83)for the entire cohort and 60 months (38–92) for event-free patients. At 60 months, 88 patients experienced KGF (CIF (95% CI): 12% (9.1–15)). At 60 months, the cumulative incidence of kidney graft failure was 11% (95% CI: 7.8–14) in patients aged <65 years and 14% (95% CI: 9–20) in those aged ≥65 years (crude sHR: 1.15, 95% CI: 0.73–1.80; *p* = 0.5) (Figure 1).

Similarly, at 60 months, the cumulative incidence was 11% (95% CI: 8.1–14) in patients aged <70 years and 16% (95% CI: 9.2–25) in those aged ≥70 years (crude sHR: 1.16, 95% CI: 0.74–1.80; *p* = 0.5).

BMI, diabetes, donor age, female donors, length of stay, and grade ≥ 3 Clavien-Dindo complications were associated with graft survival at *p* < 0.2 (Table 3).

### 3.4. Overall Survival

**Figure 2:**
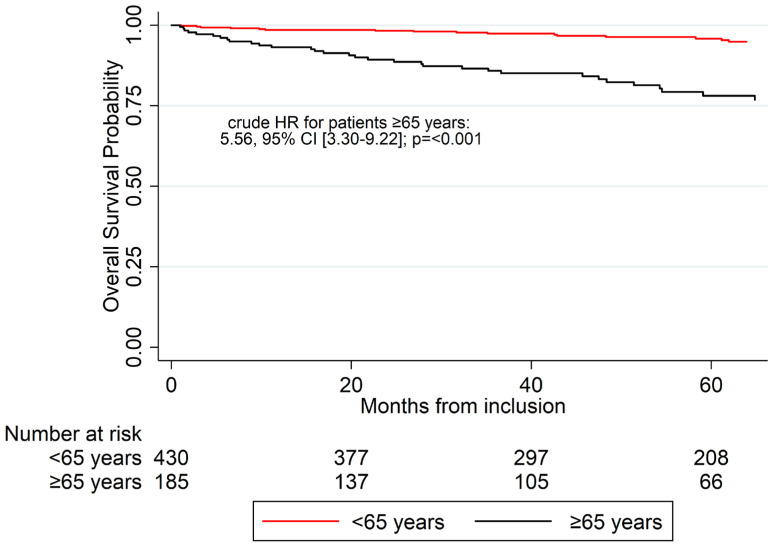
Comparison of the overall survival probabilities between patients aged ≥65 years and younger over a 60-month follow-up period. HR: hazard ratio, 95% CI: 95% confidence interval.

60-month OS probability for the entire cohort was 0.91, 95% CI (88–93). The 60-month OS was 0.95 (0.93–0.98) for patients aged <65 and 0.78 (0.70–0.84) for patients aged ≥65, respectively (crude HR: 5.56 [3.30–9.22]; *p* < 0.001) (Figure 2).

### 3.5. Multivariable Competing Risk Analysis

Candidate variables for multivariable subhazard regression analysis included: recipient sex, BMI, donor age, hypertension, diabetes, female donors, length of stay, cold ischemia time, type of donor, multiple veins, and grade ≥ 3 Clavien-Dindo complications. After adjusting for these factors, similar KGF were observed between patients aged ≥65 years (sHR: 0.75, 95% CI [0.41–1.38]; *p* = 0.36) (Table 4).

When a 70-year threshold was used, previous transplants and the donor type were also associated with patients aged ≥70 at *p* < 0.2 and included in the multivariable model (Table 2). After adjustment, similar results were observed using the 70-year threshold (sHR: 0.86, 95% CI [0.42–1.75]; *p* = 0.7) (Table 4).

## 4. Discussion

In this retrospective single-center cohort study of 618 deceased donor kidney transplants undergoing a Taguchi ureteroneocystostomy, our unadjusted analysis showed that patients aged ≥65 years had a non-statistically significant 15% higher subhazard of kidney graft failure compared with younger recipients, after accounting for death as a competing event. The effect of worse baseline characteristics, worse quality grafts, higher comorbidities, and lower overall survival in older recipients remained undetectable.

### 4.1. Studies Showing Worse Graft Survival

Our study observed similar KGF rates between older and younger recipients, contrasting with previous research reporting worse outcomes in older recipients. These discrepancies may be explained by differences in donor age, which influences post-transplant function [15]. In our study, the median age of deceased donors was 58 years (IQR: 48–67), and among recipients aged ≥65 years, the mean age was 66 years, significantly higher than that of younger recipients. In contrast, previous studies reported younger donor age distributions, with mean ages ranging from 43 to 50 years [16,17,18,19].

We observed similar results regarding KGF between older and younger recipients, whether the age threshold was set at ≥65 or ≥70 years. These results contrast with real-world data from the US, which show worse graft survival (GS) in patients aged ≥65 years compared to those aged 18–34 years (5-year OS of 68% vs. 81%, respectively) [20]. Also, a meta-analysis of four studies showed worse GS at 5 years in recipients aged >70 years compared to younger ones (RR, 1.37; 95% CI, 1.14–1.65). However, this meta-analysis observed no statistically significant differences in GS at 1 and 3 years [10].

In our cohort, recipients aged ≥70 years received kidneys from donors who were, on average, 15 years older than those allocated to younger recipients (mean donor age 68.6 vs. 53.7 years). This contrasts with the findings of Artiles et al. [10] where the difference was only 8 years (47.7 vs. 55.0 years). Our study also included a higher overall proportion of DBD, with no significant differences between older and younger recipients (81% vs. 79%). In contrast, the proportion of DBD donors in this meta-analysis was generally lower (46% vs. 18%). Furthermore, our cohort was restricted to DCD and DBD donors, whereas the meta-analysis estimators for graft survival also incorporated living donors.

Despite older recipients of our cohort having worse quality donors, these factors did not influence graft survival. Our results align with previous meta-analyses showing no impact of obesity on graft survival at 3 and 5 years [21], hypertension, diabetes, or cold ischemia time at 1 year [15]. As in our study, grade 2 or higher Clavien-Dindo complications were associated with OS and KGF in univariate analyses; however, after adjusting for other factors, this association was no longer observed [22].

The absence of difference in graft survival in our study, could be explained by the fact that patients aged ≥65 are less likely to be included in the transplantation waiting list (HR 0.80.18; 95% CI, 0.17–0.18) and to receive a first KT (HR = 0.88; 95% CI, 0.87–0.89) than younger patients [23]. Also, the increasing number of older patients on the kidney transplant waiting list over the past decade suggests that more older adults are being considered for transplantation. However, older patients are at higher risk of waitlist removal and death after waitlist removal [20]. These factors may indicate a selection bias regarding graft survival, as patients who do receive a KT may represent a subset of healthier patients who were fit enough to undergo a KT. However, it is important to note that older recipients in our cohort received grafts from older donors, experienced more complications, and were more frequently diabetic, hypertensive, and exposed to longer ischemia times. Even if healthier older patients were selectively included in our database, these worse baseline characteristics and the lower quality of grafts would be expected to negatively influence outcomes. Nevertheless, this was not the case, as graft survival remained comparable across groups.

Although our cohort included only deceased-donor kidney transplants, it is important to note that outcomes in older adults may differ in the setting of living donor transplantation. El Hennawy and colleagues reported that graft and patient survival at 1 year were comparable between groups, although by 3 years survival was lower in older recipients [24]. In another study, recipients of kidneys from donors aged 70 to 89 years with a donor–recipient age difference of −10 to 15 years had worse graft survival compared to those receiving grafts from donors aged 30 to 49 years. However, graft survival was not significantly different for recipients of donors aged 50 to 69 years, nor for those aged 70 to 89 years when the donor–recipient age difference was wider (15–40 years) [25]. These findings suggest that living donor transplantation may mitigate some of the risks associated with older age; however, differences in long-term outcomes remain.

### 4.2. Studies Showing Worse OS

In our study, recipients aged ≥65 years had worse survival outcomes than younger ones. These results have been confirmed in recent meta-analyses showing worse survival in older patients [10]. However, this difference in OS has also been observed among deceased kidney donor recipients aged 60–69 when compared to those aged >70. In Visan et al.’s study, recipients aged >70 years experienced worse OS at 3 and 5 years than (3-year OS: 63% vs. 78%, and 5-year OS: 58% and 73%, respectively) [26].

### 4.3. Strengths and Limitations

The limitations of our study include its retrospective, observational nature and single-center design, as well as its limited sample size when compared to other studies [16,19].

Variable selection for multivariable analysis was performed based on the literature and the available data in our database. However, some variables were not recorded, and unmeasured confounders such as delayed graft function and the number of HLA mismatches were not accounted for. Despite these variables having been associated with KGF in previous meta-analyses, the effect size and the degree of certainty of its effect are moderate [15].

Previous studies have shown an association between donor quality indexes such as the Kidney Donor Profile Index (KDPI) or the Kidney Donor Risk Index. Although we do not have information regarding the KDPI in our dataset, several methodological concerns have been raised regarding these and other donor quality indices [27]. For instance, multiple studies have evaluated the predictive performance of the KDPI; however, important methodological limitations of these studies can be noted, including the use of arbitrary thresholds and the absence of utility measures, such as net benefits, to assess clinical usefulness in decision-making. For example, Khan et al. [28], assessed the clinical utility of the recalibrated KDPI model and observed benefit only at survival probability thresholds above 80%. In practical terms, this implies that the model would be useful only when anticipating a graft failure probability of ≤20%. Given that such high success probabilities are already expected in most kidney transplants, the added value of applying a predictive index under these circumstances is questionable. This highlights the need for more robust and clinically meaningful donor quality metrics beyond KDPI. Moreover, donor comorbidities such as hypertension and diabetes were not recorded in our dataset. However, the impact of donor diabetes and hypertension on graft survival remains controversial, with prior studies reporting variable effect sizes [29,30,31]. Moreover, rather than donor diabetic status alone, outcomes appear to be more strongly influenced when both donor and recipient are diabetic, and by the duration of the donor’s diabetic disease, which has been associated with worse graft outcomes [32].

Another strength of our study is that, in contrast to the included studies in the Artiles and colleagues’ meta-analysis, we estimated KGF using a competitive risk analysis, rather than a conventional survival analysis or censoring death to estimate graft survival, providing less biased estimators [10,16,17,18,19]. When death is censored, the estimated probability of KGF is overestimated because patients who die before experiencing graft failure are excluded from the calculation of graft survival [14].

Also, previous studies have shown that frailty, the type and number of geriatric impairments, are associated with an increased risk of mortality and DGF in the older population with CKD [33,34,35]. Frailty may contribute to KGF through its association with DGF, an increased complication rate, and poor immunosuppressive adherence [33,36]. However, no studies specifically assessed KGF. Thus, only indirect associations regarding KGF and frailty can be established. Our study did not account for data regarding frailty measures, as our center routinely does not perform geriatric assessments due to a lack of available geriatricians; thus, the absence of frailty or geriatric-specific information could limit our results.

In our center, the selection process for transplant candidates is not determined by chronological age. Instead, we rely on a comprehensive clinical evaluation of the recipient’s overall health status and comorbidities. This assessment is individualized and does not follow a systematic scale or standardized test. Consequently, our approach may differ from prior studies that stratified candidates primarily by age or used predefined scoring systems. That said, we acknowledge that a more systematic evaluation of health status in older candidates, particularly through validated scales or structured approaches such as comprehensive geriatric assessments, could improve consistency and comparability across centers and may represent a valuable direction for future practice.

Although we have analyzed the outcomes using a unique ureteroneocystostomy technique (Taguchi), which limits heterogeneity when different surgical techniques are employed, the Taguchi reimplant is not a widely used technique and may limit the generalizability of our results. Moreover, the currently recommended ureteroneocystostomy technique is the Lich-Gregoire reimplant because it has shown better short-term results than other techniques, despite other studies not observing long-term differences between the Taguchi and Lich-Gregoire techniques [37].

### 4.4. Implications and Perspectives

Despite a higher complication and comorbidity rate among older recipients, the available evidence, though limited because of its observational nature and methodological issues, supports the benefit of transplantation in older patients, offering substantial long-term survival benefits over dialysis [8,10].

Although KT in older recipients can provide similar functional results to those in younger recipients and increase survival and quality of life compared to dialysis, KT also presents an economic challenge for health systems. For instance, KT results are cost-effective in patients aged up to 70 years with mild comorbidities, or healthy patients when the waiting list is less than 2 years [38].

## 5. Conclusions

Our findings indicate that while older kidney transplant recipients had worse baseline characteristics, had grafts of worse quality, and experienced higher rates of postoperative complications, the observed kidney graft survival remains comparable to that of younger recipients. This underscores the viability of kidney transplantation in older patients and suggests that transplant selection committees should not exclude older candidates solely based on age or comorbidities, but rather consider them viable candidates when appropriately selected.

## Figures and Tables

**Table 1:** Demographic and clinical characteristics of participants.

Variable	n (%)
Sex	
Male	412 (67%)
Female	206 (33%)
Recipient’s age, median (IQR)	58 (47, 66)
Weight (kg), median (IQR)	75 (65, 86.2)
Height (cm), median (IQR)	166 (160, 173)
Body mass index, median (IQR)	27.2 (23.9–30.8)
Type of donor	
Donors after circulatory death	120 (19%)
Donors after brain death	498 (81%)
Donor’s sex	
Male	374 (61%)
Female	236 (38%)
Donor’s age, median (IQR)	58 (48, 67)
Previous grafts	82 (13%)
Hypertension	533 (86%)
Diabetes	177 (29%)
Number of grafts	
First	536 (87%)
Second	76 (12%)
Third	6 (1%)
More than one kidney graft artery	107 (17%)
More than one kidney graft vein	10 (2%)
More than one kidney graft ureter	6 (1%)
Ischemia time (hours), median (IQR)	14 (7.5, 19)
Length of stay in days, median (IQR)	11 (9, 16)
Clavien-Dindo grade ≥ 3 complications	153 (25%)
Arterial stenosis	13 (2%)
Thrombosis	7 (1%)
Ureteral stenosis	33 (5%)
Postoperative transfusion	177 (29%)
Renal graft hematoma	46 (7%)
Postoperative hematuria	40 (6%)
BK virus infection	40 (6%)
Symptomatic Lymphocele	29 (5%)
Lithiasis	3 (<1%)
Urine leaks	19 (3%)
Wound complications	42 (7%)

**Table 2:** Comparison of patients’ characteristics between age groups using different age thresholds.

Variable	<65 Years	≥65 Years	*p*-Value	OR (95% CI)	*p*-Value	<70 Years	≥70 Years	*p*-Value
	431 (70)	187 (30)				524 (83%)	94 (15%)	
Female receptors, n (%)	153 (35%)	53 (28%)	0.1 *	0.72 (0.49–1.04)	0.08	178 (34%)	28 (30%)	0.4 *
Recipient’s age, mean (SD)	50.30 (10.06)	70.12 (4.25)	<0.001 ^µ^			53.2 (11.09)	73.49 (3.31)	<0.001 ^µ^
Body mass index, mean (SD)	27.21 (4.79)	28.02 (4.15)	0.047 ^µ^	1.04 (1–1.08)	0.047	27.25 (4.66)	28.57 (4.29)	0.01 ^µ^
Hypertension, n (%)	361 (84%)	172 (92%)	0.01 *	2.22 (1.24–4)	0.01	447 (85%)	86 (91%)	0.1 *
Diabetes, n (%)	99 (23%)	78 (42%)	<0.001 *	2.4 (1.66–3.46)	<0.001	134 (26%)	43 (46%)	<0.001 *
Previous grafts, n (%)	60 (14%)	22 (12%)	0.5 *	0.82 (0.49–1.39)	0.5	75 (14%)	7 (7%)	0.1 *
Donor’s age, mean (SD)	51.27 (13.64)	66.72 (9.47)	<0.001 ^µ^	1.13 (1.11–1.16)	<0.001	53.69 (14.12)	68.59 (7.86)	<0.001 ^µ^
Female donors, n (%)	166 (39%)	70 (38%)	0.8 *	0.95 (0.67–1.35)	0.8	202 (39%)	34 (37%)	0.6 *
Ischemia time (hours), mean (SD)	13.40 (6.28)	14.49 (6.76)	0.04 ^µ^	1.03 (1–1.06)	0.06	13.40 (6.28)	14.49 (6.76)	0.04 ^µ^
Length of stay (days), mean (SD)	13.16 (8.47)	15.99 (11.76)	<0.001 ^µ^	1.03 (1.01–1.05)	<0.001	13.68 (8.97)	15.88 (12.73)	0.04 ^µ^
DCD, n (%)	80 (19%)	40 (21%)	0.4 *	0.84 (0.55–1.28)	0.4	96 (18%)	24 (26%)	0.1 *
More than one artery, n (%)	74 (17%)	33 (18%)	0.9 *	1.03 (0.66–1.62)	0.9	93 (18%)	14 (15%)	0.5 *
More than one vein, n (%)	7 (2%)	3 (2%)	0.6 ^θ^	0.99 (0.25–3.86)	0.99	8 (2%)	2 (2%)	0.5 ^θ^
More than one ureter, n (%)	3 (1%)	3 (2%)	0.3 ^θ^	2.33 (0.47–11.63)	0.3	5 (1%)	1 (1%)	0.6 ^θ^
Clavien-Dindo grade ≥ 3 complications, n (%)	91 (21%)	62 (33%)	0.001 *	1.85 (1.26–2.72)	<0.001	122 (23%)	31 (33%)	0.045 *

^µ^ T-student test, * Chi-squared test, ^θ^ Fisher’s exact test, OR: Odds Ratio, CI: Confidence Interval, SD: Standard Deviation, DCD: Donors After Circulatory Death.

**Table 3:** Analysis of Factors Influencing 60-Month Overall Survival and Kidney Graft Failure.

	60-Month Overall Survival		60-Month Kidney Graft Failure	
Variable	Crude Hazard Ratio (95% CI)	*p*-Value *	Crude Subhazard Ratio (95% CI)	*p*-Value ^µ^
≥65 years	5.56 (3.35–9.22)	<0.001	1.15 (0.73–1.80)	0.5
Female receptors	0.73 (0.43–1.25)	0.4	0.98 (0.63–1.51)	0.9
Body mass index	1.06 (1.00–1.11)	0.03	1.04 (1.00–1.08)	0.1
Hypertension	2.16 (0.87–5.38)	0.7	1.11 (0.59–2.09)	0.7
Diabetes	2.22 (1.36–3.61)	0.001	1.36 (0.87–2.12)	0.2
Previous grafts	1.11 (0.55–2.25)	0.8	1.06 (0.55–2.01)	0.9
Donor’s age	1.06 (1.03–1.08)	<0.001	1.03 (1.02–1.05)	<0.001
Female donors	1.14 (0.69–1.87)	0.6	1.61 (1.05–2.48)	0.03
Ischemia time (hours)	1.02 (0.98–1.06)	0.5	1.02 (0.98–1.05)	0.3
Length of stay (days)	1.05 (1.03–1.06)	<0.001	1.03 (1.01–1.04)	<0.001
Donors After Circulatory Death	0.36 (0.20–0.63)	<0.001	1.36 (0.70–2.65)	0.4
More than one artery	1.16 (0.62–2.17)	0.6	0.81 (0.44–1.49)	0.5
More than one vein	3.47 (0.84–14.30)	0.1	2.01 (0.46–8.78)	0.4
More than one ureter	1.13 (0.16–8.16)	0.9	1.87 (0.57–6.16)	0.3
Clavien-Dindo grade ≥ 3 complications	2.16 (1.13–3.56)	0.003	2.30 (1.49–3.45)	<0.001

* *p*-value from the Wald test derived via Cox regression analysis; ^µ^ *p*-value from the Wald test derived via a Fine and Gray regression analysis; CI: Confidence Interval.

**Table 4:** Multivariable competing risk analysis of factors influencing 60-month kidney graft failure using different age thresholds.

	≥65 Years Threshold		≥70 Years Threshold	
Variable	Adjusted Subhazard Ratio (95% CI)	*p*-Value ^µ^	Adjusted Subhazard Ratio (95% CI)	*p*-Value ^µ^
Older patients	0.73 (0.41–1.29)	0.3	0.79 (0.40–1.55)	0.5
Female receptors	1.06 (0.67–1.67)	0.8	1.05 (0.67–1.66)	0.8
Body mass index	1.03 (0.98–1.08)	0.2	1.03 (0.98–1.08)	0.2
Hypertension	1.17 (0.57–2.41)	0.7	1.17 (0.56–2.47)	0.7
Diabetes	1.07 (0.60–1.89)	0.8	1.04 (0.59–1.81)	0.9
Previous grafts	-	-	0.92 (0.42–2.01)	0.8
Donor’s age	1.02 (1.00–1.04)	0.04	1.02 (1.00–1.04)	0.1
Female donors	1.72 (1.09–2.73)	0.02	1.75 (1.10–2.78)	0.02
Ischemia time (hours)	1.00 (0.96–1.04)	0.9	1.00 (0.96–1.04)	0.9
Length of stay (days)	1.02 (1.00–1.04)	0.04	1.02 (1.00–1.04)	0.04
Donors After Circulatory Death	1.28 (0.64–2.58)	0.5	1.27 (0.63–2.58)	0.3
More than one vein	2.49 (0.49–12.55)	0.5	2.51 (0.49–12.97)	0.3
Clavien-Dindo grade ≥ 3 complications	1.57 (0.90–2.73)	0.1	1.56 (0.89–2.72)	0.1

^µ^ *p*-value from the Wald test derived via a Fine and Gray regression analysis; CI: Confidence Interval.

## Data Availability

The data supporting the findings of this study are available from the corresponding author upon reasonable request.
